# The Challenges to Promoting Attachment for Hospitalised Infants with NAS

**DOI:** 10.3390/children8020167

**Published:** 2021-02-22

**Authors:** Jaylene Shannon, Kath Peters, Stacy Blythe

**Affiliations:** 1 Generalist Community Nursing, Mid North Coast Local Health District, Wauchope 2446, Australia; Jaylene.Shannon@health.nsw.gov.au; 2 School of Nursing and Midwifery, Western Sydney University, Penrith 2750, Australia; k.peters@westernsydney.edu.au

**Keywords:** neonatal abstinence syndrome, attachment, nurses, midwives, NAS, NICU, special care nursery

## Abstract

The postnatal period is crucial for infants in establishing a connection with and security in primary caregivers and can have enduring effects on attachment patterns. However, due to the need for symptom management, many infants diagnosed with neonatal abstinence syndrome (NAS) may be separated from primary caregivers and cared for in a neonatal intensive care unit (NICU) or special care nursery (SCN) soon after birth. Research has shown that substance-exposed infants are more likely to experience insecure attachment patterns with their primary caregivers and that mothers with a history of substance abuse are less sensitive to their infants’ cues. Therefore, the aim of this research was to explore nurses’ and midwives’ experiences in promoting the attachment relationship for infants admitted to an NICU/SCN with NAS. A qualitative research design was used to gather data on the experiences of nine nurses/midwives from various NICU and SCN settings in Australia. Individual, semi-structured interviews were conducted, and transcribed interviews were coded using thematic analysis. While nurses/midwives valued the attachment relationship for infants with NAS, facilitation of the attachment relationship was mainly promoted when the mother was present. However, parents were often reported to be absent from the nursery. Difficulties in promoting an attachment relationship were also identified when an infant had child protection involvement. This research identifies areas in need of innovative change regarding the approach taken to promote the attachment relationship for infants with NAS when they are admitted to an NICU/SCN.

## 1. Introduction

With the increases in opioid abuse during pregnancy and the additional use of multiple concurrent licit and illicit substances during pregnancy, neonatal abstinence syndrome (NAS) has become significantly more common and more complex to treat, placing significant economic, social and healthcare burdens on society [1]. Pharmacological intervention for symptom management is required in up to 91% of neonates with NAS and requires admission to a special care nursery (SCN) or neonatal intensive care unit (NICU) for monitoring [2]. This outcome separates the infant from the mother during a crucial period of infant development, bonding and establishment of the attachment relationship. Early work conducted by Klaus and colleagues (1972) identified the benefits that extended close contact in the postpartum period has on infant–mother attachment [3]. Labour and birth is associated with hormonal and instinctive responses in the mother and infant that aid in the promotion of the initial bonding experience [4]. Immediate and uninterrupted skin-to-skin contact after birth is associated with enhanced bonding over the weeks and days following birth, decreases difficulties with breastfeeding and increases the likelihood of a secure attachment relationship at 12 months postpartum [4]. Bystrova and colleagues (2009) identified the lasting impact that immediate postnatal mother–infant separation has on the mother–infant relationship even after one year postpartum [5]. Mothers who experienced immediate contact with their infants after birth and experienced rooming-in during their hospital admission were likely to display more maternal sensitivity and responsiveness to their infants after 12 months compared to mothers whose infants were separated from them soon after birth and were only taken to their mothers during feeding times [5]. 

Poor attachment in infancy and early childhood is known to correlate with the development of mental health disorders. Up to 20% of children and adolescents worldwide will experience mental health disorders [6]. Without treatment, mental health disorders negatively impact children’s ability to live a fulfilling and productive life and result in significant societal costs in terms of healthcare, social services and legal support [7]. While there is increasing awareness of the implications of mental illness in children and adolescents, there is a general lack of recognition of the impact that mental illness has during infancy (birth–3 years) [7]. This is concerning, as developmental studies have long recognised that early infancy is a period of rapid brain and behavioural development which places the individual in a vulnerable position within his or her environment [8,9,10,11]. The period of infancy is unique in that there is a complete dependence on a primary caregiver as central to the infant’s world and their ability to regulate emotional and physiological needs. This dependency on a caregiver leaves the infant particularly vulnerable to the stress responses that the caregiver endures in their environment and can have lasting effects on the infant’s own development of stress responses [7]. The quality of the caregiver’s attentiveness and sensitivity to the infant’s needs is essential in regulation development and has epigenetic implications affecting brain growth and stress system function [12,13]. Therefore, infants may benefit from treatment within the infant–caregiver relationship model. 

There is a lack of research focused on promoting attachment for infants with NAS. In particular, this includes a paucity of research into the experiences of NICU and SCN nurses/midwives. During the postnatal period, these clinicians are in a unique position to facilitate the attachment relationship between the mother and infant [14]. With the knowledge that attachment is enhanced by close proximity of the infant to the mother and the responsivity of the mother to her infant’s cues, nurses/midwives can focus nursing/midwifery interventions on facilitating this process [14]. Therefore, nurses/midwives play an integral role in facilitating the attachment relationship between a mother and her infant during the postnatal period. The aim of this study was to contribute to the literature by exploring the experiences of registered nurses and/or midwives who have cared for infants experiencing NAS and how they have supported the development of an attachment relationship for these infants.

## 2. Materials and Methods

### 2.1. Design of the Study

The natural phenomena within this study’s context are unique and complex; therefore, a qualitative research design based on naturalistic inquiry was the elected methodology. Naturalistic inquiry refers to the process of investigating and preserving the natural experiences of study participants in order to discover new knowledge that has emerged from real experiences [15]. Special care (SC) and neonatal intensive care (NICU) registered nurses/midwives (RN/RM) were interviewed to gain an understanding of their individual experiences within their natural workplace settings and how they have promoted the attachment relationship for NAS infants within these settings.

### 2.2. Recruitment

Participants were recruited between April and September 2017 via a poster that was emailed to various contacts and colleagues of the research team and by asking those contacts to distribute the poster to others who may be interested in participating in the study. Participants were included in the study if they (1) were a registered nurse or midwife and (2) had cared for an infant experiencing symptoms of NAS in the SCN or NICU. All RNs and RMs who volunteered and who had experienced caring for infants experiencing NAS were included in the study. 

Participants were also encouraged to pass details of the study on to their colleagues who they believed may also be interested in participating in the research as a snowballing technique for recruitment [16]. 

### 2.3. Data Collection 

Staying true to naturalistic inquiry, the researcher was the human instrument for data collection by conducting semi-structured individual interviews [17]. These interviews were conducted by the first author, an experienced qualitative interviewer, using either face-to-face (*n* = 1), online (*n* = 1) or telephone (*n* = 7) methods. Multiple methods allowed individual interviews to be negotiated with each participant and reduced inconvenience and limitations to location and accessibility for researchers and participants [18]. Interviews lasted between 20 and 49 min, with an average of approximately 30 min, and consisted of 7 open-ended questions. This style of interview provided a structured format for the researcher by predetermining open-ended questions to maintain focus on the topic being explored [18]. 

### 2.4. Data Analysis 

After each interview, the researcher wrote a reflective account of the interview, analysing thoughts, feelings and processes of the interview in order to remain unbiased and to note key information that was a focus in the individual interviews. The researcher also made small notes while conducting the interviews. This “field journal” is a strategy often used in naturalistic inquiry which allows the researcher to remain reflexive throughout the data collection process [19]. 

Interview data were transcribed into a Word document using a professional transcription service. When transcripts were returned to the researcher, the transcripts were read and re-read several times before line-by-line coding was applied. This was performed by highlighting sentences or phrases and using the comment tool in Microsoft Word to apply a code. No limit to the number of codes was set and codes were determined by using a word or short phrase based on what the dialogue was representing. This form of manual thematic analysis ensured that all data were meticulously analysed to allow accuracy and rigour [20].

Codes were then displayed without the transcripts and like-phenomena were grouped together, which allowed conceptualisation of sub-themes to be developed [21]. The coded transcription text was then grouped together into these themes through the use of a table. By referring back to the transcribed dialogue, adjustments could be made to ensure that the participant’s transcribed dialogue was being represented accurately by the sub-themes and grouped appropriately. This was done in consultation with all authors. By organising data with this method, conclusions were able to be efficiently drawn [20].

After data were thoroughly refined, it was possible to summarise the findings. These findings were confirmed by looking back at the raw data in the form of recorded interviews and transcribed dialogues and establishing the significance of the conclusions. During each of the data analysis stages, especially as data were being coded, the researchers checked for consistency by selecting random pages of the transcripts and re-coding them.

Participants in the study described experiences that occurred in different hospitals with differing circumstances, allowing a broad overview of differing situational experiences that may occur in any other hospital setting. Participants were continually recruited into the study until interviews reached the point when no new information was being gathered and data became repetitive. 

### 2.5. Ethical Considerations

Approval to conduct this research was granted by the Western Sydney University Human Research Ethics Committee, approval number H12098. All participants provided both written and verbal consent prior to the interviews. In order to maintain privacy and confidentiality, transcripts were deidentified. Excerpts of transcripts portrayed throughout the findings were allocated identifiers (e.g., RN#). 

## 3. Results

### 3.1. Description of the Study Participants

Participants included both RNs and RMs from hospitals in New South Wales, Victoria and the Northern Territory of Australia, including large metropolitan public and private hospitals and smaller remote/regional public hospitals. Nine participants in total were purposively selected and included one male registered nurse/midwife, two female registered nurse/midwives and six registered nurses. The lowest level of qualification of participants was a Bachelor’s degree, with most participants having a post-graduate certificate and the highest level of education being a PhD. Participants were aged between 30 and 65 years old and had from 4 to 45 years of clinical experience in nursing. One participant came from a culturally and linguistically diverse (CALD) background and spoke English as a second language. Experience with neonates ranged between 3 and 25 years. One participant was no longer working clinically in nursing/midwifery but had remained up to date with contemporary practices on neonatal nursing and NAS. One participant was working as the Nurse Unit Manager of a special care nursery and one participant was working as a Nurse Unit Manager in another specialty field of nursing unrelated to neonatal nursing. The remaining six participants were working within an SCN or NICU at the time of interview.

### 3.2. Themes

Analysis of the transcribed interviews revealed three major themes: (i) Facilitating the attachment relationship; (ii) barriers to promoting attachment; and (iii) complexities associated with care for hospitalised infants with NAS. Each of these major themes had sub-themes. Given the depth of the data, this paper reports findings from the first two major themes only (Table 1). The findings revealed in the third major theme are presented in another paper within this Special Issue.

#### 3.2.1. Facilitating the Attachment Relationship

The benefits to the infant—“Attachment’s quite vital to their outcomes.”

Participants believed that promoting and establishing an attachment relationship for an infant with NAS was important for these infants, mainly for the benefits they believed attachment had on the physical outcomes of care. Participants observed that infants who appeared to have some form of attachment to their primary caregivers were able to feed better, put on weight and were able to be weaned off medication sooner due to the infant’s temperament changing to become more settled.

“I think if it’s done right and done consistently then NAS would—there’d be less symptoms of it. I think that what I saw was a baby settled better, fed better, and certainly grew, put on weight, developed, reached its milestones, maybe not right on when they should, but certainly reached them than compared to those who didn’t go through attachment responses. So I think all in all it was a positive thing.”

**RN 1**

“The benefits are, well they settle better, they feed better, they have a routine, if the parents are attached well…. The baby settles, it puts on weight, it feeds better and is generally just a more settled baby, even though it might be being treated with opiates for withdrawal.”

**RN 8**

The participants’ main focus for the benefits of attachment was on short-term physical outcomes. Participants believed that the major benefits of promoting attachment were that the length of hospital stay was reduced because infants were able to be weaned off pharmacological management sooner.

“I think attachment in the nursery for a baby always reduces the length of stay and enhances effective treatment.”

**RN 8**

“I’m sure the more attachment they have the length of stay is often reduced because they become more settled. You can wean medication a lot quicker and attachment with their [parent is]—In the long-term I think the best measure of successful attachment is length of stay. I think sometimes they may stay longer than—I think that the discharge time would be reduced so that we’d actually get babies home earlier if they were treated with the parents.”

**RN 6**

Interestingly, only one participant reflected on the importance of long-term impacts that establishing an attachment relationship can have on the infant. The participant described the distressing symptoms that NAS produces and reflected on the impact that this sort of distress may cause for the infant if they are not soothed and comforted by someone consistently. The participant reported that this may have some sort of negative impact on the child in the long term.

“I think it’s important because attachment is extremely important for NAS baby because the baby constantly will not settle. Withdrawal syndrome, the baby does need someone reliable to be there when he or she has a need to be calm. We don’t know when we don’t facilitate the attachment when the baby is in the middle of withdrawal syndrome what kind of negative impact to this child when he or she grow up.”

**RN 5**

While the participants mainly focused on the short-term physical benefits to the infant, the improvement to parental interaction was also observed by participants. Participants believed that parents of infants experiencing NAS would often come into the NICU/SCN more frequently if they had begun to establish an attachment relationship with their infant and were more confident in the way they handled their infant and understood the infant’s symptoms.

“I think a parent who is attached well to their baby handles their baby differently to someone who’s not attached, and visits like once every now and again, whereas someone who visits regularly has a bit of a routine.”

**RN 8**

Participants believed that establishing an attachment relationship was vital to the care given to infants with NAS. However, they identified many barriers to promoting the attachment relationship within the NICU/SCN setting for infants with NAS.

#### 3.2.2. Barriers to Promoting Attachment

The symptoms of NAS—“Mothers sometimes find it hard to attach to the baby because they’re screaming all the time.”

Nurses found that due to the nature of the symptoms of NAS, these babies can have difficulty forming an attachment relationship to their caregivers. Participants recognised the extra challenge for the mother as the infant has extra needs that would be complex for the mother to deal with on her own.

“…because at the end of the day they are a really high needs child and they can get quite challenging when they’re withdrawing and quite inconsolable.”

**RN 9**

“I think it’s—it must be awfully traumatic to have a baby that you just can’t do anything with. You can cuddle, cuddle and cuddle and feed, and feed and feed, and they just won’t settle.”

**RN 3**

Participants recognised that the presentation of the symptom of withdrawal as a high-pitched cry would be a particularly stressful symptom for a mother to soothe and feel a connection to her baby.

“It’s very difficult I think, it would be from the mother’s point of view, to attach to a baby who’s screaming all the time, and just won’t settle.”

**RN 3**

It was also recognised that parents do not always understand the nature of the symptoms of NAS and may misinterpret the extreme agitation of the infant as the parent not having the ability to comfort the infant or the infant not liking them.

“So I think often mothers sometimes find it hard to attach to the baby because they’re screaming all the time and they take it personally that the baby doesn’t like them or that it’s something—they’re not holding them right or whatever rather than it’s just a symptom of their withdrawal.”

**RN 7**

Biological parents’ unique circumstances—“Mothers of NAS babies need that extra support.”

When the symptoms of NAS become extreme and the infant requires pharmacological management as a result, it is common practice for infants to be transferred to an NICU or SCN for additional monitoring and administration of medication. This results in the infant’s subsequent separation from the mother. Participants recognised that it would be extremely difficult for parents and infants to establish an attachment relationship, not only due to the symptoms of NAS but also due to the physical separation from their baby during this acute period. This is conveyed in the following dialogue:

“It would be very difficult for a mum to attach to her baby when it’s away from her. But, oh, I don’t know—it’s—it—I just would find it very difficult for any sort of attachment, especially in those first few days when the baby’s at its worst.”

**RN 3**

Participants recognised the complex social and family context that infants that experience NAS can often be born into. Many participants believed that multiple confounding factors of the biological parents’ lifestyle impacted the ability for nurses and midwives to promote attachment. The most significant barrier the participants recognised was the availability of the parents in the NICU/SCN. When parents were not present in the NICU/SCN, the nurses felt that they could not assist parents with establishing the attachment relationship with their infant.

“We often find that if they’re not actually present, they don’t attach to the babies very well. It’s hard and they seem to be a bit—a lot more distant from them.”

**RN 6**

Participants recognised that, often, this was due to difficulties with transport, as parents of infants with NAS often have a lower socio-economic background and often do not have a car and rely on public transport or cannot afford to pay for things such as parking and petrol.

“She didn’t have a car so she couldn’t get in very often. She depended on rides in the cab and the taxi.”

**RN 4**

“Usually by this stage they are discharged so it means they have to make their way into the hospital and often they don’t have transport, and they find it hard to pay for parking or bus fares and things like that. So it makes it difficult for some parents to actually come in and be with their babies.”

**RN 7**

Child protective services—“They couldn’t find anywhere for him to go.”

Participants recognised the difficulty in promoting an attachment relationship for infants experiencing NAS when a family has child protection services involved. In some cases, the parents still have access to the child, but this may be restricted while family services are involved.

“Sometimes the parents have restricted visiting because the baby might already be identified as at risk and under the care of community services who stipulate when the parents can and can’t visit or it’s supervised visits only with a case worker.”

**RN 2**

Participants described the difference in the way the mother would interact with her infant if she only had occasional visitation rights while the infant was in the NICU/SCN.

“You would always see the mother come in when she was allowed to visit and the baby would become agitated again. That’s because she didn’t have the confidence, or she was squeezing baby too tight because she just wanted to keep with that baby. I don’t know.”

**RN 1**

Participants recognised the disruption to the development of attachment that occurs for these infants that have been removed from their biological mother.

“It has happened a couple times. Like babies that are—like you said, that are separated legally from the mothers, and the mother actually can’t come in and nobody is coming in. That’s—there’s been one baby like that in the nursery when I was there, so it actually had nobody.”

**RN 4**

“If they’ve been absolutely relinquished or the parents have still got visitation rights, or if they’re just not going home with the parents but they’ve still got visitation rights, sometimes that becomes quite complex. So it becomes a sort of time of detachment and the foster mother still doesn’t really know the baby, so you get no real attachment.”

**RN 8**

Participants recognised that infants experiencing NAS who had child protection involvement were often missing out on a lot of the benefits that an attachment relationship provides, such as comfort and feelings of security, as the nursing staff were often too busy to provide this. This is portrayed in the following dialogue:

“I suppose that’s often the problem with these babies whose parents either don’t visit or they’ve totally been removed and can’t visit; that they miss out on that normal infant bonding things of being held and you cry and someone picks you up and all that kind of thing because the nurses are busy.”

**RN 7**

In the absence of primary caregivers and the high workloads of nurses/midwives, participants revealed that infants with NAS were often left to cry.

“But it seemed like after a while he kind of would give up, if we couldn’t come and if he was crying but I had four other babies to feed or whatever. It just—sometimes I felt sorry for him, because I just thought he just gave up.”

**RN 4**

Participants in this study recognised this as an early sign of insecure attachment.

“So we realised that he hadn’t bonded with anybody and because he was that bit older he cried and didn’t actually expect anyone to come.”

**RN 7**

One participant discussed the differences between the promotion of attachment in the presence and absence of the parent. This participant described how attachment strategies are well integrated into practice when parents are there; however, when parents are no longer involved, attachment is almost forgotten about.

“I think sometimes we’re missing it sometimes. So the babies are missing out on that opportunity. I don’t know why we’re not transferring that across the board. Like yep, birthing unit, baby’s born. Skin on skin, wrap that blanket around, stay there, but different and if we’re not—we don’t have a lightbulb blinking sometimes to remind us yes, this infant has been removed from mum but don’t forget, how can we ensure that we don’t sort of—that this doesn’t go by the wayside, that we don’t miss an opportunity sort of thing—particularly I think NAS babies I think are dealt a [short] straw.”

**RN 2**

Participants painted a picture showing that practices in hospitals are initiated to promote maternal bonding, but in the absence of a maternal or caregiving figure, the development of an attachment figure for the child is no longer considered.

“I think we just assume—or I think a lot of people assume that it’s just about mum and that if mum can’t be there, oh well that’s a shame but we did the best we can sort of thing. Not if we don’t get this right, what are the implications for this child as opposed to this infant kind of thing, that it’s not just limited to this neonatal period or for the first three months of this infant’s life.”

**RN 2**

Participants recognised the importance of forming an attachment to the mother even if the infant would be removed from the mother’s care. They believed that the benefits of an established attachment was better than no attachment at all.

“Even if baby is not going home to mum, if mum is able and willing to visit then that baby coming out and developing an attachment to mum although will be discontinuous and the baby won’t be going to mum’s care, developing an attachment to her and having that discontinue, baby will transfer that attachment to whoever becomes their next carer but if we don’t do that in that initial phase and wait three months until the carer goes—until the child goes home, we’re missing a really important step.”

**RN 2**

Challenges to finding the appropriate foster care homes for infants was recognised by participants and often meant that NAS infants had no appointed caregiver to form an attachment relationship to for a significant period of time.

“But also too we had a little Aboriginal boy who was going into care and he was an early baby but he also needed oxygen, and had all these other medical problems and things like that. We realised—so he was a couple—or nearly three months corrected and he was still in the nursery because they couldn’t find anywhere for him to go because they were trying to place him with an Indigenous family, but no one could smoke in the household.”

**RN 7**

Often, when the infant is still unwell, participants reported that foster carers are not appointed until the baby reaches a stage when they are physically well enough to go home.

“So they will find a foster family as soon as they can, unless the baby’s sick of course, and there are other issues, but if it’s a basically well baby, then, yeah, they’ll find a carer.”

**RN 3**

It was evident that nurses found it difficult to promote attachment relationships for infants when it was finally determined that they would be going into foster care. Often, infants would remain in the nursery until they were placed, and when a foster parent was appointed, the child would be discharged quickly as the infant was medically well. Participants reported that they would not always get the opportunity to promote the attachment relationship between the foster parent and the infant.

“I found it really challenging when the baby was going into foster care but [Child Protective Services] were taking the baby directly so we didn’t really get to interact with the carer themselves, or have the carer get to know the baby.”

**RN 9**

In the absence of the Biological Parent—“I guess we just do what we can.”

Participants recognised that in the absence of the parent from the NICU/SCN, the attachment relationship was a lot more difficult for nurses and midwives to promote due to the essential role that the parent has in the attachment relationship. When the parents still had custody of their child but could not attend the NICU/SCN very often, participants did become concerned about the establishment of attachment for infants. This is conveyed in the following dialogue:

“Yeah, so I was a bit concerned about the baby’s attachment with the mother because the mother couldn’t come in as often as would have been good. So I think that’s probably why I felt a bit more attached to the baby, because I knew that he wasn’t getting what he needed from his mother.”

**RN 4**

When parents were frequently absent from the NICU/SCN, it was often the nurses and midwives who had to fulfil the role of the attachment figure.

“I remember on night duty holding those babies myself. I couldn’t do skin-to-skin, but we had the babies close and you keep them swaddled and essentially they can hear your heartbeat and smell the smells and hear those natural noises that were around.”

**RN 1**

“So it was kind of up to us to give him comfort when he was having all that distress.”

**RN 4**

Participants reported that hospital volunteers were a valuable strategy to ensure that infants experiencing NAS were given the opportunity to feel soothed and comforted by someone, but usually, these were only utilised if the infant had child protection issues.

“We also have—we do have older ladies that come in and volunteer, and they actually sit and cuddle babies. So we’ll call on them if we need to, if the baby has nobody, they’re just lying there all day with nobody to cuddle besides us.”

**RN 4**

Participants reported that night duty was particularly difficult as hospital volunteers were not present and less staff were available to provide comfort for NAS infants.

“But then you had a problem overnight, because they [volunteers] weren’t there overnight. So we’d always rely on the actual nurse to be able to do that and the staffing didn’t always allow that. So you sometimes had a really crappy night trying to deal with the unsettled baby, with the other ones, so that made it hard.”

**RN 1**

It was also difficult when hospital volunteers were not always available as they had days off or were being utilised elsewhere in the hospital. Participants observed that this lack of consistency had negative implications for the infants.

“They would tend to go backwards. So you’d get this baby into a routine and then all of a sudden there’s no one around to hold them, or to swaddle them, or to just soothe them.”

**RN 1**

## 4. Discussion

The aetiology of NAS is multifactorial, and while promoting attachment may improve outcomes, there are multiple confounding factors that impact on the long-term outcomes for infants who have experienced NAS [21]. However, the importance of attachment for infants with NAS is slowly gaining traction as more and more infants are hospitalised for this condition [22,23,24]. This paper adds to the body of literature on NAS by highlighting the unique challenges nurses/midwives experience in promoting attachment for some infants with NAS. In particular, parental absence from the nursery due to child protection involvement, stigma and social circumstances proved to be a considerable barrier to their ability to promote attachment. Although nurses/midwives have a role in promoting attachment, they are not the primary attachment figure. Therefore, it is not their role to form an attachment relationship with the infant but rather facilitate this with the infant’s parents. Without the presence of a primary caregiver, however, it is impossible for nurses/midwives to facilitate this attachment. This highlights a crucial gap in practice where the absence of a parent results in the dilemma of the infant not being attached to anyone. 

In the current study, attachment was recognised by nurses/midwives as vital for infant development and as an important aspect for effective parenting. However, when nurses/midwives described the benefits of promoting an attachment relationship, they focused on the physiological outcomes for the infant. Specifically, they focused on the reduced need for pharmacological management of symptoms and decreased length of hospital stay rather than on the psychological and developmental outcomes for the infant. Only when it was recognised by the participants that the promotion of attachment to the mother would reduce the treatment needs of the infant with NAS, and result in a faster discharge, did the priority of promoting an attachment relationship to the mother become a higher priority. Therefore, it is plausible to say that the attachment relationship was mostly valued as a physiological symptom management strategy for infants with NAS. This finding resonates with previous discussions in the literature [24]. 

Because physiological benefits and treatment outcomes were seen as the motivation for the promotion of the attachment relationship, when child protection services were involved and the infant with NAS was removed from the mother’s care, the promotion of an attachment relationship was no longer perceived as valid due to the absence of a caregiver. This leaves the infant at even greater risk for an insecure attachment. Phillips (2013) highlighted the mechanism of the infant cry as a communication of distress or discomfort to the mother [25]. The instinctive response within the mother is to bring the infant close to her body for protection and warmth. However, in the instance that the infant’s crying does not produce the intended response, the infant’s instinctive biological response will be to quieten and become still as a defence adaptation to minimise attention from potential predators [26]. In the absence of the mother or another appointed primary caregiver, nurses/midwives in the current study described heart-breaking moments when they would witness an infant with NAS “give up” and cease crying as nobody was able to attend to them. Phillips (2013) refers to this moment as “despair” in the infant [25]. In this state, the infant’s body systems will slow down to prolong survival as the infant is largely dependent on the mother for warmth, protection and nutrition [25]. It is therefore not surprising that the participants in the current study noticed the physiological benefits that the presence of the mother and the promotion of the attachment relationship had on the physiological outcomes for infants. However, attachment relies heavily on an infant’s emotional needs being met, and a primary caregiver’s ability to respond quickly and appropriately to the infant’s communication of distress is fundamental for establishment of a secure attachment relationship [14]. When the communicated needs of the infant are met, the infant develops trust and security [14]. Consistent failure to meet these needs results in the development of insecure attachment styles [14] and predisposition to life-long mental health issues. 

The attachment relational system is fundamental to the development of neurotransmitter activity and response to stress in early infancy [7]. Prolonged distress leads to long-term elevated cortisol levels that do not typically decrease, even after the factor producing the distress is no longer there [27]. Elevated cortisol levels have been documented in attachment studies of infants with disorganised attachment patterns after experiencing only mild stressors [28]. Infants who experience disorganised attachment relationships are more likely to struggle with emotional regulation, aggression and social isolation and display behavioural issues in childhood and mental illness in adulthood [29]. The current study has revealed that the NICU/SCN environment for infants with NAS is likely contributing to the development of insecure attachments for infants with NAS, rather than promoting a secure attachment, due to competing priorities of nursing/midwifery care and the focus on physiological well-being over psychological well-being.

The findings from this study can be used to guide future research. It identifies areas in need of change in the approach to promoting the attachment relationship for infants with NAS when they are admitted to the NICU or SCN, so that parents of an infant experiencing NAS can be given every opportunity to enhance the formation of the attachment relationship with their infant. Changes to the environment where infants requiring pharmacological treatment for NAS are cared for may also enhance the attachment relationship by allowing rooming-in of parents with their infant. Rooming-in studies have demonstrated positive outcomes for NAS infants including decreased length of hospital stay, decreased need for pharmacological management of symptoms, improved rates of breastfeeding and decreased involvement of child protection services [30,31,32,33]. Key elements to the success of these studies included using a family-focused model of care that partnered with caregivers by providing staff education of the illness of NAS and the needs of parents of these infants.

There is also a need to research strategies that can be implemented for infants who have child protection involvement and who have been removed from the custody of their parents. The transition from the custody of the parent to the foster carer was not straightforward for nurses/midwives, and the promotion of the attachment relationship for the infant was difficult for nurses/midwives while waiting for an appointed foster carer. Often, there was no opportunity for nurses/midwives to support the foster carer and infant with establishing an attachment relationship. Therefore, clear processes are required that aim to minimise the disruption of the attachment relationship for infants with NAS. Such strategies may include assignment of a cuddle-mum, prompt assignment of a foster carer who can visit regularly and training for foster carers and cuddle-mums on promoting the establishment of the attachment relationship.

Changes to current guidelines need to be made to highlight the impact that the disrupted attachment relationship has on an infant with NAS who has child protection involvement. No recommendations are made for how to reduce the impact on the infant’s attachment relationship when this occurs. Strategies that nurses/midwives and other hospital staff can utilise during the admission to the NICU/SCN need to be identified so that staff can minimise the risk to the infant. Nurses/midwives are responsible for providing holistic care, and therefore, they need to be empowered to promote the attachment relationship for all infants.

Development of assessment tools that nurses/midwives could use as evidence-based practice in the nursery setting may be beneficial for assessing the level of risk an infant has for the development of an insecure attachment and ongoing assessment of strategies that may be used to minimise this risk for infants. Research could be aimed at measuring the risks and measuring the success of interventions used to reduce the risks for infants with NAS in the NICU and SCN.

An identified barrier to the promotion of the attachment relationship for infants with NAS was parental absence. Even when child protection services were not involved in the custody of the infant, nurses/midwives found that parents were frequently absent from the NICU/SCN. Changes to the hospital setting to minimise this barrier to promoting attachment may include changing the setting where infants requiring pharmacological management of NAS are cared for, such as keeping infants on the maternity ward, or transfer to the paediatric ward where rooming-in can be offered. Another option would be to provide special training and employ a surrogate carer within the NICU/SCN who specialises in promoting the attachment relationship for NAS infants and families and can establish a working relationship with the family to assist mothers/caregivers with the promotion of the attachment relationship. When the mother cannot be present in the nursery, the surrogate carer could be there to ensure that the infant is still cuddled and soothed and could ensure that a smooth transition is offered to foster carers if the infant is removed from the custody of the mother. In the current study, nurses/midwives identified that volunteer cuddle-mums were a valued resource for these infants. However, these cuddle-mums were not always available and did not have special training in NAS or attachment theory. Furthermore, the use of multiple, different volunteers and the lack of consistency are not conducive to establishing an attachment relationship.

This qualitative study is the first of its kind, specifically looking at the attachment relationship for infants with NAS and how this is promoted in the postnatal period by nurses and midwives. Qualitative studies are contextual in nature, and therefore, the findings are not generalisable. Despite the small sample size, the rich data and descriptions presented here enable readers to consider transferability to similar settings. 

## 5. Conclusions

While nurses/midwives valued the attachment relationship for infants with NAS, facilitation of the attachment relationship could only be promoted when parents or caregivers were present. However, parents were often reported to be absent. Parental absence in the NICU/SCN was a major barrier for promoting the attachment relationship and meeting the psychological and emotional needs of the infant. In the absence of parents, nurses/midwives found themselves struggling to meet the holistic aspects of care regarding the emotional and social needs of the infant, particularly when the infant had child protection involvement. This research has highlighted the differing holistic needs of infants with NAS in comparison to other infants admitted to the NICU/SCN. Furthermore, this current study has identified the need for innovative change regarding the approach taken to promote the attachment relationship for infants with NAS when they are admitted to the NICU/SCN. Without substantial changes to policies and practice regarding the delivery of care to infants with NAS, unacceptable risk to the well-being of these infants throughout their life trajectories remains.

## Figures and Tables

**Table 1:** Summary of main themes and sub-themes from interview data.

Main Theme	Sub-Theme
Facilitating The Attachment Relationship	The benefits to the infant—*“Attachment’s quite vital to their outcomes”*
Barriers to Promoting Attachment	The symptoms of NAS*—“Mothers sometimes find it hard to attach to the baby because they’re screaming all the time”*
	Biological parents’ unique circumstances—*“Mothers of NAS babies need that extra support”*
	Child protective services—*“They couldn’t find anywhere for him to go”*
	In the absence of the mother—*“ I guess we just do what we can”*

## Data Availability

The data presented in this study are available on request from the corresponding author. The data are not publicly available due to privacy restrictions.
